# Functional role of histone variant Htz1 in the stress response to oleate in *Saccharomyces cerevisiae*

**DOI:** 10.1042/BSR20150114

**Published:** 2015-07-07

**Authors:** Hongde Liu, Guanghui Li, Lingjie Liu, Yakun Wan

**Affiliations:** * State Key Laboratory of Bioelectronics, Southeast University, Nan jing 210096, China; † The Key Laboratory of Developmental Genes and Human Disease, Ministry of Education, Institute of Life Sciences, Southeast University, Nanjing 210096, China; ‡ Jiangsu Nanobody Engineering and Research Center, Nantong 226010, China

**Keywords:** Htz1, nucleosome, oleate stress

## Abstract

Chromatin structure is implicated in regulating gene transcription in stress response. Transcription factors, transferases and deacetylases, such as multicopy suppressor of SNF1 protein 2 (Msn2), SET domain-containing protein 1 (Set1) and sucrose NonFermenting protein 1 (Snf1), have been identified as key regulators in stress response. In the present study, we reported the dynamics of nucleosome occupancy, Histone Two A Z1 (Htz1) deposition and histone H3 lysine 4 dimethylation (H3K4me2) and histone H3 lysine 79 trimethylation (H3K79me3) in *Saccharomyces cerevisiae* under oleate stress. Our results indicated that citrate cycle-associated genes are enhanced and ribosome genes are repressed during the glucose-oleate shift. Importantly, Htz1 acts as a sensor for oleate stress. High-throughput ChIP-chip analysis showed that Htz1 has redistributed across the genome during oleate stress. The number of Htz1-bound genes increases with stress and the number of Htz1-bound ribosome genes decreases with stress. The dynamics of Htz1 and H3K79me3 around transcription factor-binding sites correlate with transcriptional changes. Moreover, we found that nucleosome dynamics are coupled with Htz1 binding changes upon stress. In unstressed conditions (2% glucose), nucleosome occupancy is comparable between Htz1-bound genes and Htz1-depleted genes; in stressed conditions (0.2% oleate for 8 h), the nucleosome occupancy of Htz1-depleted genes is significantly lower than that of Htz1-bound genes. We also found that Msn2 acts an important role in response to the oleate stress and Htz1 is dynamic in Msn2-target genes. Htz1 senses the oleate stress and undergoes a global redistribution and this change couples dynamics of nucleosome occupancy. Our analysis suggests that Htz1 and nucleosome dynamics change in response to oleate stress.

## Abstract (precis)

In response to oleate stress in *Saccharomyces cerevisiaes*, Histone Two A Z1 (Htz1) undergoes a global redistribution during the glucose-oleate shift. The number of Htz1-bound genes increases, but the number of Htz1-bound ribosome genes decreases with stress. Citrate cycle-associated genes are enhanced and ribosome genes are repressed. Nucleosome dynamics are coupled with Htz1-binding changes upon stress. Multicopy suppressor of SNF1 protein 2 (Msn2) acts an important role in response to the oleate stress. We highlight the dynamics of Htz1 in the oleate stress.

## BACKGROUND

Chromatin structure is implicated in the regulation of gene transcription in stress responses [1–3]. Many transcription regulators were identified in stress response, including the transcription factors Msn2 (multicopy suppressor of SNF1 protein 2) and Msn4, the lysine methyl transferase Set1 (SET domain-containing protein 1), the histone deacetylase Rpd3 (Reduced potassium dependency protein 3) and Snf2. Moreover, the roles of the regulators in the stress response are closely associated with nucleosome organization, the distribution of histone modifications and histone variants.

Msn2 and Msn4 both act in response to environmental stress [4]. Elfving et al. [5] suggested a dynamic interplay between nucleosomes and Msn2 in which nucleosomes can restrict the accessibility of Msn2 whereas Msn2 can promote the re-organization of nucleosomes to alter expression. Also, the histone deacetylase Rpd3 links with Msn2 under stress [6]. Histone H3 lysine 4 (H3K4) methylation primarily acts as a gene repressor during multiple stresses, specifically at genes involved in ribosome biosynthesis [1]. Under transcriptional stress, H3K4 trimethylation (H3K4me3) is shifted to the 3′-end of the gene *translation elongation factor 1 (TEF1)*, whereas nucleosomes are deposited at 3′ positions of genes [2]. The repression of ribosome genes is frequently suggested in stress [7]. Acetylation of *SNF2* is suggested in the recruitment of Swi/Snf for stress-responsive genes, including ribosome genes [7,8]. Histone Two A Z1 (Htz1), a variant of histone H2A, is frequently found at two transcription start site (TSS)-vicinity promoters. Zanton and Pugh [9] suggested that the assembly of active transcription complexes in heat stress is coupled with the eviction of Htz1-containing nucleosomes and disassembly is coupled with the return of nucleosomes.

The studies described above indicated that chromatin dynamics are important in stress responses. Moreover, the functions of many regulators are strictly linked to chromatin. In the present study, we report our studies of *Saccharomyces cerevisiae* under oleate stress. We focused on the dynamics of nucleosome occupancy, Htz1 deposition and the modifications H3K4 dimethylation (H3K4me2) and histone H3 lysine 79 trimethylation (H3K79me3). We intend to provide some clues to several questions. First, what is the functional role of Htz1 in response to stress? Although Htz1 deposition in chromatin has been studied extensively [3–9], what is Htz1 dynamics upon stress and how the dynamics affect nucleosome occupancy and gene expression remain unclear. Second, are H3K4me2 and H3K79me3 binding patterns modified in response to stress? Because H3K4me3 and its transferase, Set1, are suggested to have a role in the stress response, we wanted to know if the distribution of H3K4me2, which is also methylated by Set1 [10], is similar to that of H3K4me3. H3K79me3, which mainly covers the gene body, is correlated with gene expression [11]. We examined how genomic binding of H3K79me3 changes upon stress. Third, what is the stress consequence in response to oleate in yeast? Oleate represents a mild alteration in carbon source. Oleate exposure can disturb lipid and membrane homoeostasis and alter phospholipid composition and the formation of an additional lipid class, ethyl esters of fatty acids, in yeast [12]. We are interested in the chromatin dynamics elicited by oleate.

## MATERIALS AND METHODS

### Yeast strains and growth conditions

The yeast strains used in this study are listed in Supplementary Table S1. A PCR-based, one-step gene modification method was used for the genomic integration of genes encoding C-terminal HA (influenza virus hemagglutinin) fusions [13]. Yeast transformations were performed using a lithium acetate/PEG-based method [14]. Strains were cultured at 30°C in the following media: YPD (1% yeast extract, 2% peptone, 2% glucose; unstressed condition), SCIM [0.17% yeast nitrogen base without amino acids and ammonium sulfate (YNB-aa-as), 0.5% yeast extract, 0.5% peptone, 0.079% complete supplement mixture, 0.5% ammonium sulfate] containing 0.5% Tween 40 (w/v) and 0.2% oleate (w/v) for 8 h (stressed condition).

### ChIP

For each ChIP experiment, strains were grown first in glucose medium (YPD) to a density of 1×10^7^ cells/ml and then transferred to oleate medium (SCIM) to grow for 8 h. ChIP was performed as described above. In brief, sheared chromatin lysates were incubated with antibody-conjugated magnetic beads overnight at 4°C with rotation. Following incubation, cross-links were reversed in both the ChIP and the whole-cell lysate fractions and samples were analysed by DNA tiling microarrays [15].

### ChIP-chip

For genome-wide analysis of Htz1, Pol II (RNA polymerase II), H3K4me2 and H3K79me3, ChIP was performed as described above. Linkers were annealed to the ends of the ChIP and input whole cell extract (WCE) DNA samples and DNA was then amplified by PCR. The amplified DNA from the IP and input samples were labelled using an RNA Fluorescent Labeling DNA kit (Kreatech). The labelled ChIP and input DNA were hybridized to *S. cerevisiae* whole-genome tiling arrays (4×44 k; Agilent). Data were read using Agilent Feature Extraction software.

### Nucleosome positioning analysis

Wild-type cells were grown in YPD medium to a *D*_600_ of 1.0 and transferred to SCIM medium for 8 h. All cells were then treated with formaldehyde and incubated with 125 mM glycine. Cell permeabilization, micrococcal nuclease digestion, protein degradation and DNA purification steps were performed as previously described [16]. DNA samples were treated with RNase A and separated on a 2% agarose gel to assess the nucleosomal content. Bands corresponding to mono-nucleosomal DNA were extracted using a Qiagen Gel Extraction kit (Qiagen). Mono-nucleosomal DNA libraries were prepared and sequenced using an Illumina Genome Analyzer II (Illumina Inc.) according to the manufacturer's instructions.

### Co-ordinates of transcription start and termination sites and transcription factor-binding sites

The genomic co-ordinates of both ends of 5419 transcripts were retrieved from the *Saccharomyces* Genome Database (SGD; http://www.yeastgenome.org/). The transcription rate data of yeast genes were obtained in a previous study [17]. The dataset containing the regulation relationship of transcription factors and their target was previously published [18]. Only the transcription factor–target pairs that associate with a *P*-value less than 10^−3^ were used. TFBSs (transcription factor-binding sites) data were previously published (http://younglab.wi.mit.edu/regulatory_code/) [19]. The expression data in heat shock and nitrogen source depletion stresses is retrieved from literature [20].

### Htz1, Pol II, H3K4me2 and H3K79me3 profiles

The profile of any genomic region that is covered by a probe is represented by the log_2_-binding ratio of the probe; the profile of a region that is not covered by a probe was set to zero. An Htz1-bound gene has at least one Htz1-bound probe that was identified within the region of −500–+300 bp of the TSS. Htz1-bound probes were identified using two criteria: (1) the central probe's binding ratio is >1 and the *P*-value is <0.05 and (2) the *P*-value of either of the two neighbouring probes is <0.25. The Pol II-bound genes, the H3K4me2-associated genes and the H3K79me3-associated genes were identified similarly except that, for the two latter cases, the region of interest is the gene body [+300 bp to TTS (transcription termination site)].

### Nucleosome occupancy data analysis

The raw reads of sequencing for nucleosome occupancy were mapped on to the *S. cerevisiae* strain S288c genome using Bowtie [21]. Only the uniquely mapped reads were used in further analysis. First, the length of each read was extended 73 bp in the 3′ direction and the Waston-strand reads and Crick-strand reads were oppositely shifted 73 bp. The absolute nucleosome occupancy value of each genomic site was expressed as the number of reads covering the genomic sites. Second, the nucleosome occupancy was scaled by dividing values by the average nucleosome occupancy of the whole genome, i.e., the nucleosome occupancy was expressed as the fold change of the absolute occupancy relative to the average occupancy.

The nucleosome occupancy profile near the special sites (TSSs and TFBSs) were represented by the average nucleosome occupancy profile, which was calculated by summing the occupancy signal at each genomic site and then dividing the summed signal by the gene number [22]. The dyad coordinates of nucleosomes were determined with a wavelet transformed-based algorithm (the ‘peaksfind ()’ function in Matlab).

### Enrichment analysis of the Kyoto Encyclopedia of Genes and Genomes pathway and Gene Ontology

Enrichment analysis was carried out for Kyoto Encyclopedia of Genes and Genomes (KEGG) pathway and Gene Ontology (GO) term data for Htz1-bound, Pol II-bound, H3K4me2-associated and H3K79me3-associated genes using the functional annotation table module of DAVID (http://david.abcc.ncifcrf.gov/). For a selected GO term, we calculated the *P*-value absolute difference {Δ[−log10(P)=−log10(P)_chz1Δ − log10(P)_WT]} between the unstressed and the stressed strains to identify the change in the enrichment significance.

## RESULTS

### Htz1 binding on chromatin correlates with gene transcription changes between unstressed (wild-type in 2% glucose) and oleate-stressed strains

We determined the binding of Htz1 and Pol II on chromatin and the modifications of H3K4me2 and H3K79me3 with ChIP-chip experiments and we determined nucleosome occupancy by next generation sequencing in unstressed and oleate-stressed strains. Figure 1(A) shows the nucleosome, Htz1 and Pol II profiles for 5419 genes that are sorted by their transcription rate. As expected [23,24], nucleosomes are depleted near TSSs and form an array with equal spacing downstream of the TSS. Htz1 binding is mainly enriched at the promoter, especially at the −1 and +1 nucleosome and near TTSs. Increased Pol II binding is observed for the genes with a high transcription rate. We calculated the Pearson correlation coefficients (*r*) of the log_2_-binding ratio of all probes for pairs of Htz1, Pol II, H3K4me2 and H3K79me2 (Figure 1B). Compared with the Pol II, H3K4me2 and H3K79me3 profiles, the Htz1 binding shows less correlation (*r*=0.76) between the unstressed and stressed cells, suggesting that Htz1 binding undergoes a global change in response to oleate stress. Htz1 binding is slightly negatively correlated with H3K4me2 and positively correlated with H3K79me3 (Figure 1B). If there is at least one bound probe at the promoter (−500 bp ~ +300 bp of TSS) of a gene, we defined the gene as an Htz1 promoter-bound gene. Similarly, we defined Htz1 gene body-bound genes. Gene body means from+300 bp to TTS region. We found that the number of Htz1 promoter-bound genes increased 3-folds after stress (top table of Figure 1C). However, this number for Pol II, H3K4me2 and H3K79me3 showed a smaller change. Figure 1(C) also shows that both H3K4me2 and H3K79me3 mainly cover the gene body.

**Figure 1:**
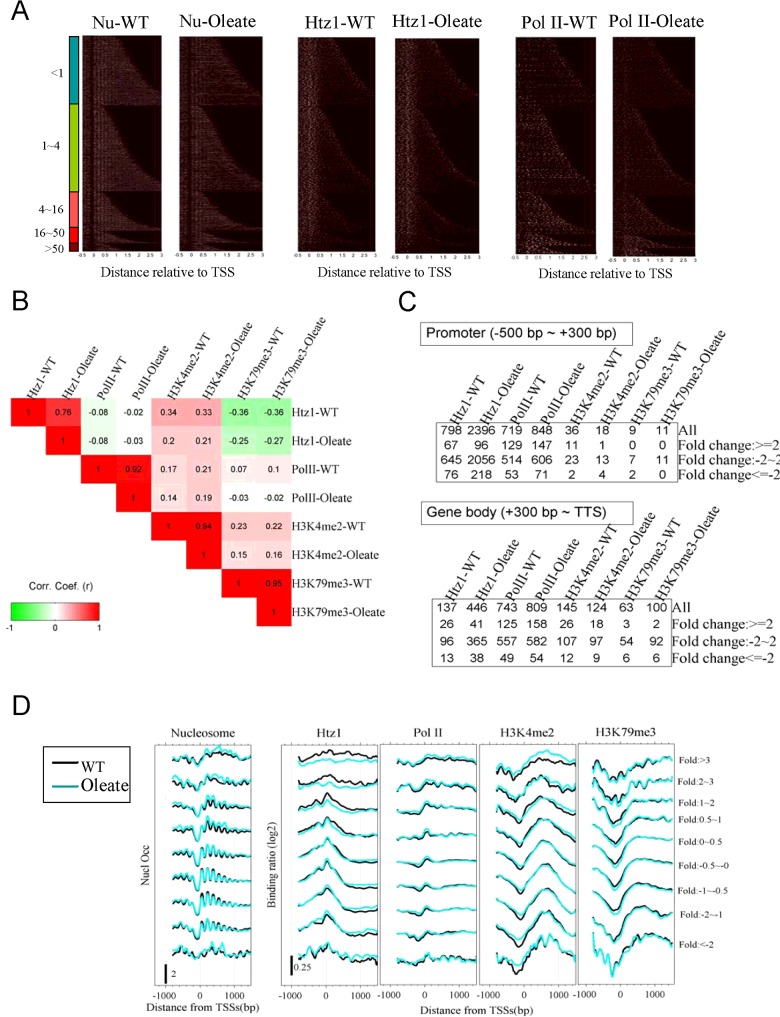
Changes in Htz1 binding in response to oleate stress (**A**) Nucleosome occupancy and Htz1 and Pol II binding profiles in unstressed and oleate-stressed cells. Yeast genes (5419) are separated into five classes according to their transcription rate in the unstressed condition: frequency < 1, 1 ≤ frequency < 4, 4 ≤ frequency < 16, 16 ≤ frequency < 50 and frequency ≥ 50. In each class, the genes were sorted by length. (**B**) Pearson correlation coefficients (*r*) of the log_2_-binding ratio of all probes for Htz1, Pol II, H3K4me2 and H3K79me3 between unstressed and oleate-stressed cells. The Htz1 correlation between unstressed and oleate-stressed cells is low (0.76), indicating that Htz1 binding changes with the stress. (**C**) The number of genes that were bound by Htz1 and Pol II and that were associated with H3K4me2 and H3K79me3 modifications in the promoter and gene body was determined. If there is at least one bound (associated) probe in a specific region of a gene, the gene is counted. A bound (or associated) probe was defined as a probe with a binding ratio > 2 and a *P*-value < 0.05 and the *P*-value of either of the two neighbouring probes was < 0.25. The binding ratio refers to the ratio of the hybridization values for Cy3 [protein (Spt16, Htz1 and Pol II)-bound DNA] compared with Cy5 (genomic DNA). (**D**) Nucleosome occupancy profiles, log_2_-ratio of Htz1 and Pol II and log_2_-ratio of H3K4me2 and H3K79me3 for the genes with differential expression between the unstressed and oleate-stressed cells.

We wanted to know the effect of the five chromatin regulators on gene expression upon stress; therefore, we plotted the nucleosome occupancy, Htz1, Pol II, H3K4me2 and H3K79me3 profiles of the unstressed and stressed conditions for genes with different fold change of expression (Figure 1D). Interestingly, the genes with a dramatic expression change (fold change > 2 or < −2) show a broader nucleosome-depleted region near TSSs in both conditions than genes with less expression change. Up-regulated genes (fold change > 2) are associated with decreased Htz1 binding in the stress condition, which is consistent with our previous study [15]. Pol II stalls near the +1 nucleosome of the genes that are with less expression change upon stress. In the up-regulated genes (fold change > 2), Pol II covers the downstream regions of TSSs and has increased binding with the stress. H3K4me2 increases in the gene body with oleate stress.

To investigate nucleosome dynamics in response to the stress, we classified the nucleosome dynamics as stable occupancy, position shift, occupancy increase, occupancy decrease, gain and loss by comparing the nucleosome occupancy and position between the unstressed and stressed conditions (Supplementary Figure S1A). The number of stable nucleosomes is significantly lower in up-regulated genes than in down-regulated genes in both the promoter and the gene body (Supplementary Figure S1C).

We also investigated the relationship between transcription rate and the nucleosome occupancy, Htz1 binding, Pol II binding and the H3K4me2 and H3K79me3 modifications at promoters (average value in −0.5 kbp–+0.3 kbp; Supplementary Figure S2). Pol II is positively associated with the transcription rate and Htz1 and H3K79me3 are negatively associated with the transcription rate in both unstressed and stressed conditions (Supplementary Figure S2A). However, the relationship curves do not exactly coincide between unstressed and stressed conditions (Supplementary Figure S2B). It seems that the equivalent binding of Pol II in the unstressed condition has more mRNA products than in the stressed condition; the equivalent transcription rate corresponds to less Htz1 binding in the stressed condition than in the unstressed condition (Supplementary Figure S2B).

Overall, Htz1 binding and nucleosome occupancy undergo a global change in response to the stress and the change is associated with transcription changes between the unstressed and oleate-stressed cells.

### Citrate cycle-associated genes are up-regulated and ribosome genes are down-regulated with oleate stress

We carried out enrichment analysis for 1083 up-regulated genes (fold change > 2) and 512 down-regulated genes (fold change < −2) using KEGG pathways with DAVID (Figure 2A; Supplementary Figures S3 and S4). The up-regulated genes are enriched in citrate cycle, pyruvate metabolism and other metabolism-related pathways (Figure 2A). The down-regulated genes are ribosome genes and nt metabolism-related genes (Figure 2A). This suggested that, in response to the altered carbon source, ATP-producing-related genes are up-regulated and ribosome genes are down-regulated. We also found that the up- and down-regulated genes are short in gene length (Supplementary Figure S3B). Both our results and previous findings suggested that ribosome genes are repressed in stress [1–7].

**Figure 2:**
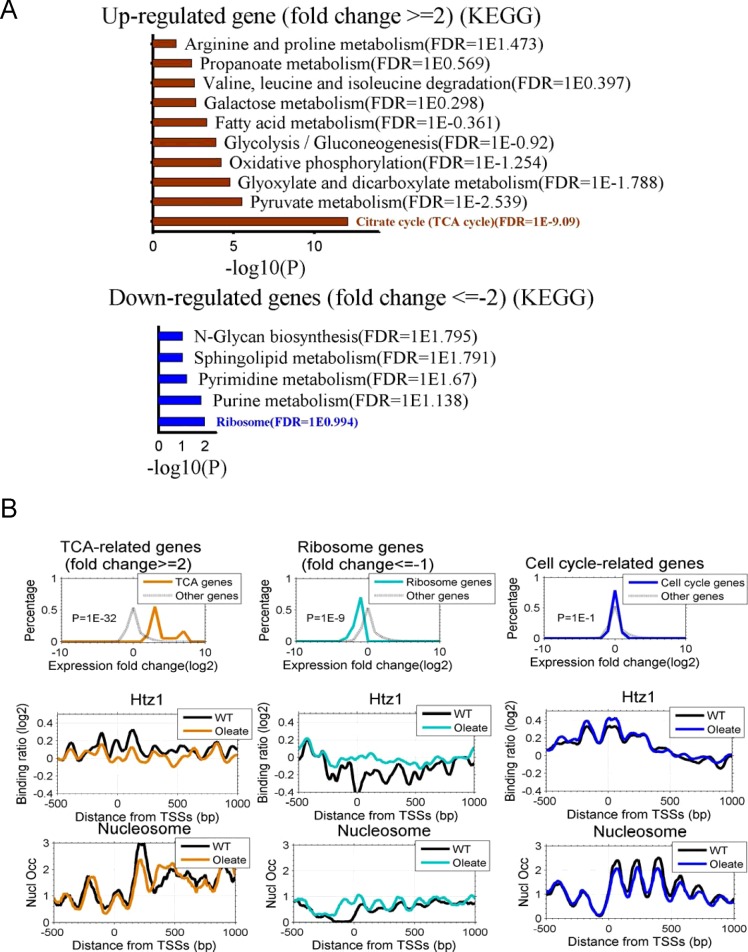
Citrate cycle-related genes are up-regulated and ribosome-related genes are down-regulated in oleate stress (**A**) KEGG pathway enrichment analysis of up- (fold change > 2; 1083 genes) and down-regulated (fold change < 2; 512 genes) genes in oleate. (**B**) Nucleosome occupancy and Htz1 binding profiles of citrate cycle-related genes, ribosome-related genes and cell cycle-related genes in unstressed and oleate-stressed cells. Three sub-plots in the top row indicate the expression distribution of the three classes of genes.

We compared the significance of GO enrichment for highly expressed genes between the unstressed and stressed conditions (Supplementary Figure S3C). Consistent with the KEGG pathway enrichment, the significance (*P*-value) of ribosome-related GO terms is greatly decreased in the stressed condition relative to the unstressed condition. The significance of ‘glucose catabolic process’ and ‘plasma membrane enriched fraction’ increases with stress.

In the above analysis (Figure 1), nucleosome occupancy and Htz1 binding change with stress. We plotted the nucleosome occupancy and Htz1 binding profiles for citrate cycle and ribosome genes (Figure 2B). The fold change of citrate cycle genes is more than two and that of ribosome genes is less than one. As expected, Htz1 binding decreases near TSSs of citrate cycle genes and increases near TSSs of ribosome genes in the stressed condition. The nucleosome occupancy of citrate cycle genes does not show an obvious change with and without stress. However, the nucleosome occupancy of ribosome genes (Figure 2B) increases near TSSs, forming two nucleosomes. This indicates a close correlation between expression down-regulation and nucleosome occupancy of ribosome genes.

We were also interested in cell cycle genes, but we did not observe any obvious changes in expression, Htz1 binding and nucleosome occupancy in oleate-stressed cells relative to unstressed cells (Figure 2B).

### Htz1 binding of Msn2/Msn4 target genes

Msn2 and Msn4 are stress-responsive transcription factors [5–25]. To investigate if the activity of these two transcription factors changes in response to oleate, we analysed the Htz1 binding and nucleosome occupancy of Msn2 and Msn4 and their target genes. We retrieved the target genes from the previously published reports [19]. First, we found that the expression of Msn2 does not greatly change with oleate stress (fold change of Msn2 is −1.1 and that of Msn4 is −2). The nucleosome occupancy of Msn2 increases at ~+300 bp upstream of the TSS in the stressed condition, indicating the gain of a nucleosome (Figure 3A). The profiles of other chromatin regulators, Htz1, Pol II, H3K4me2 and HK79me3, did not change around the promoter. Htz1 binding dramatically decreases at the promoter region of Msn4 and nucleosome occupancy decreases at ~+200 bp upstream of the Msn4 TSS (Figure 3A). Pol II, H3K4me3 and H3K79me3 show no change (Figure 3A). Next, we calculated the Htz1, Pol II and nucleosome occupancy profiles for up-regulated target genes of Msn2 and Msn4 (Figure 3B). Information of Msn2/4 target genes is from literature [19]. There are 369 and 105 up-regulated target genes of 2568-Msn2 target genes and 376-Msn4 target genes respectively. Figure 2(B) shows the Htz1 binding profiles of the genes. Obviously, Htz1 binding of the up-regulated genes is decreased upon stress. Pol II binding is slightly increased. The nucleosome occupancy remains unchanged at the promoter but increases on the gene body. These results indicate that some Msn2 and Msn4 target genes are up-regulated by oleate stress. Moreover, changes in Htz1 binding are co-ordinated with this expression change.

**Figure 3:**
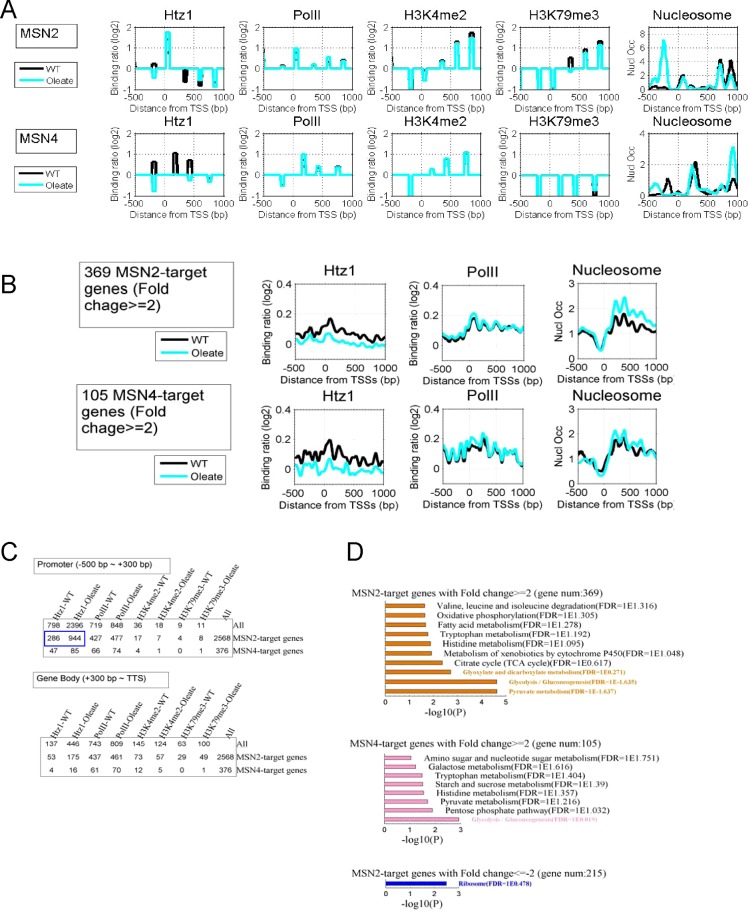
Decreased Htz1 binding is associated with the up-regulation of both Msn2 and Msn4 target genes (**A**) Nucleosome occupancy, Htz1 binding, Pol II binding, H3K4me2 and H3K79me3 profiles of the Msn2 and Msn4 genes. (**B**) Htz1 binding profiles for the up-regulated Msn4 target genes and the up-regulated Msn2 target genes in unstressed and oleate-stressed cells. Nucleosome occupancy and Pol II binding profiles are also shown. (**C**) The number of Msn2 target and Msn4 target genes that were bound by Htz1 and Pol II and that were associated with of H3K4me2 and H3K79me3 respectively. (**D**) KEGG pathway enrichment analysis of the up-regulated Msn4 target genes and Msn2 target genes and the down-regulated Msn2 target genes.

Interestingly, Htz1 binding of other Msn2 target genes increased. The number of Htz1 promoter-bound genes increased from 286 in unstressed cells to 944 in stressed cells, indicating that Htz1 is involved in the regulation of Msn2 target genes (Figure 3C). Finally, we performed a KEGG pathway enrichment analysis for up- and down-regulated Msn2 and Msn4 target genes (Figure 3D). The up-regulated Msn2 and Msn4 target genes are enriched in pathways of pyruvate metabolism and glycolysis and the down-regulated Msn2 target genes are enriched in ribosome genes. This result is consistent with the analysis of all regulated genes (Figure 2A). The down-regulated Msn4 target genes are not significantly enriched for any pathway.

Taken together, Htz1 binding is dynamic in Msn2 and Msn4 target genes during stress. The up- and down-regulated target genes of Msn2 and Msn4 are enriched similarly as other up- and down-regulated genes of this analysis. Therefore, we inferred that Msn2 and Msn4 play important roles in respond to oleate and that Htz1 association regulates their target genes.

We also compared the up- and down-regulated genes in three stress conditions: oleate, nitrogen depletion and heat shock [20] (Supplementary Figures S5A and S5C). More genes overlapped from the oleate stress and nitrogen depletion than the oleate stress and heat shock. Nucleosome occupancy, Htz1, Pol II, H3K4me2 and H3K79me3 profiles for up-regulated genes in nitrogen depletion and heat shock are shown in Supplementary Figures S5(B) and S5(D). A previous study suggested that genes that are up-regulated in nitrogen depletion are clustered and associated with nucleosome eviction in both promoters and coding regions [26]. Our results indicate that different gene groups will respond to different stresses.

### Nucleosome occupancy is significantly lower in Htz1-depleted genes with oleate stress

Because of the intimate association between Htz1 deposition and nucleosome occupancy at the promoter, we investigated the association between Htz1 binding and nucleosome occupancy upon stress. We classified 5419 yeast genes into two classes, Htz1-bound genes and Htz1-depleted genes, according to their Htz1 binding level (see ‘Materials and Methods’). An Htz1-bound gene has at least one Htz1-bound probe. All other genes are Htz1-depleted genes. Similar definitions were used for Pol II-bound, H3K4m2-associated and H3K79me3-associated genes. Figure 4(A) lists the number of Htz1-bound, Pol II-bound, H3K4me2-associated and H3K79me3-associated genes and their overlap between the unstressed and stressed conditions. There are 1761 genes that have increased Htz1 binding at the promoter with stress (Figure 4A), suggesting a great change in the Htz1 binding loci on chromatin. Htz1-bound and Htz1-depleted genes identified in the unstressed condition do not show significant expression changes (Supplementary Figure S6A), but Htz1-bound and Htz1-depleted genes identified in the stressed condition have significant expression changes (Supplementary Figure S6B). With oleate stress, Htz1-bound genes show a distinct KEGG pathway enrichment relative to that of cells from the unstressed condition (Supplementary Figure S6C).

**Figure 4:**
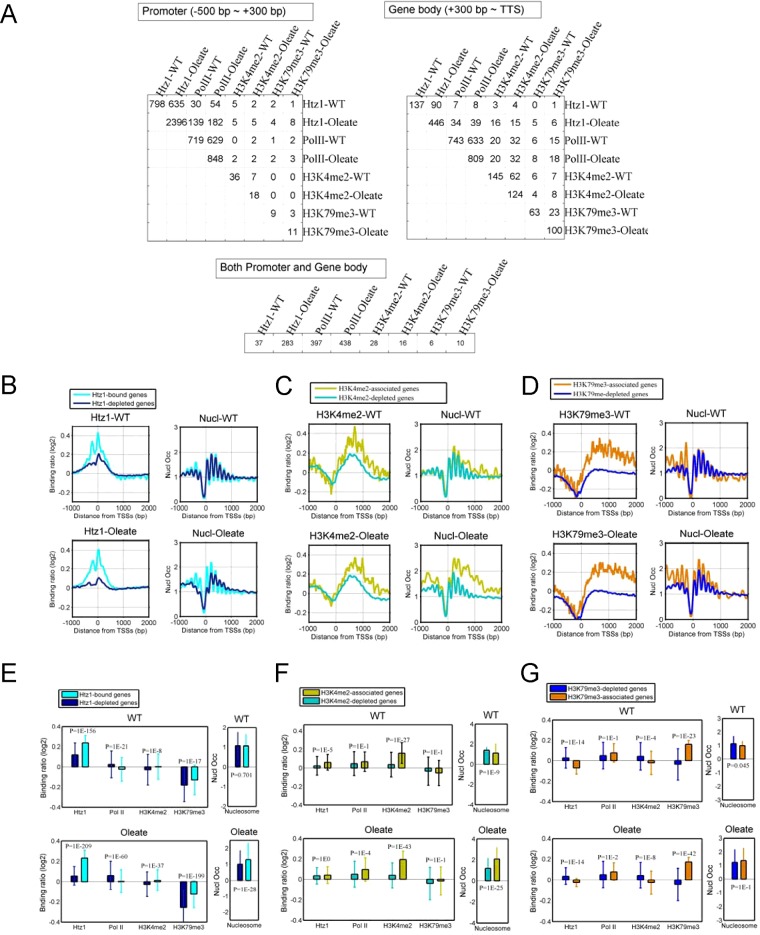
Association of Htz1, Pol II, nucleosome occupancy, H3K4me2 and H3K79me3 in oleate stress (**A**) The number of genes that were bound by Htz1 and Pol II and that were associated with H3K4me2 and H3K79me3. The intersection of the table indicates the number of overlapping genes. Further explanations are given in Figure 1(C). (**B**) Htz1 binding and nucleosome occupancy profiles of Htz1-bound genes and Htz1-dpeleted genes in the unstressed and oleate-stressed cells. If an Htz1-bound probe is identified in the promoter (−500 bp–+ 300 bp relative to theTSS) of a gene, the gene is an Htz1-bound gene. Other genes are Htz1-depleted genes. The total number of genes in the study is 5419. (**C**) Htz1 binding and nucleosome occupancy profiles of H3K4me2-associated genes and H3K4me2-dpeleted genes. If an H2K4me2-associated probe is in the gene body (+300 bp relative to the TTS) of a gene, the gene is an H3K4me2-associated gene. (**D**) Same as in (**C**) except for H3K79me3-associated genes and H3K79me3-dpeleted genes. (**E**) Average log_2_-ratio of Htz1, Pol II, H3K4me2 and H3K79me3 and the average nucleosome occupancy for Htz1-bound and Htz1-depleted genes. The *P*-value was calculated by the *t*test. The nucleosome occupancy is significantly lower in Htz1-depleted genes in oleate-stressed cells than in unstressed strain cells (wild-type; *P*=1×10^−28^). (**F**) Same as (**E**) except for H3K4me2- associated and H3K4me2-depleted genes. (**G**) Same as E except for H3K79me3- associated and H3K79me3-depleted genes.

We investigated the nucleosome occupancy of Htz1-bound genes, H3K4me2-associated genes and H3K79me3-associated genes in both unstressed and stressed conditions (Figure 4). Importantly, in the unstressed condition, the genes show a similar nucleosome occupancy profile. However, in oleate, Htz1-depleted genes show dramatically decreased occupancies at the −1 and +1 nucleosomes compared with Htz1-bound genes (Figure 4B). The average nucleosome occupancy of −500 bp–+300 bp indicates a significant difference between Htz1-bound and Htz1-depleted genes in the oleate stress condition (*P*=1×10^−28^, *t*test); but in the unstressed condition, the difference is not significant (*P*=0.701, *t*test). We hypothesized that this is a temporary chromatin state due to oleate stress.

For H3K4me2-associated and H3K4me2-depleted genes, nucleosome occupancy is not significantly changed with oleate stress (Figures 4C and 4F); the same is true for H3K79me3-associated and H3K79me3-depleted genes (Figures 4D and 4G).

### Htz1 and H3K79me3 dynamics around TFBSs correlate with transcriptional changes of downstream genes

Finally, we investigated the distribution of the five chromatin regulators near TFBSs. TFBS co-ordinates and transcription factor targets were retrieved from the literature [18,19]. Figure 5(A) shows the nucleosome occupancy, Htz1, Pol II, H3K4me2 and H3K79m3 profiles in both the unstressed and the stressed conditions. In both conditions, as expected, nucleosomes are depleted in the vicinity of TFBSs; nucleosomes are positioned on both sides of TFBSs (Figure 5A). Corresponding to nucleosome depletion, the H3K4me2 and H3K79me3 modifications are also depleted near TFBSs. The Htz1 binding signal is low near TFBS and peaks at the −200 bp and the +200 bp loci of TFBSs (Figure 4A). Pol II is enriched in the 5′ vicinity of TFBSs. We observed that Pol II binding increased at TFBSs upon stress (Figure 5A).

**Figure 5:**
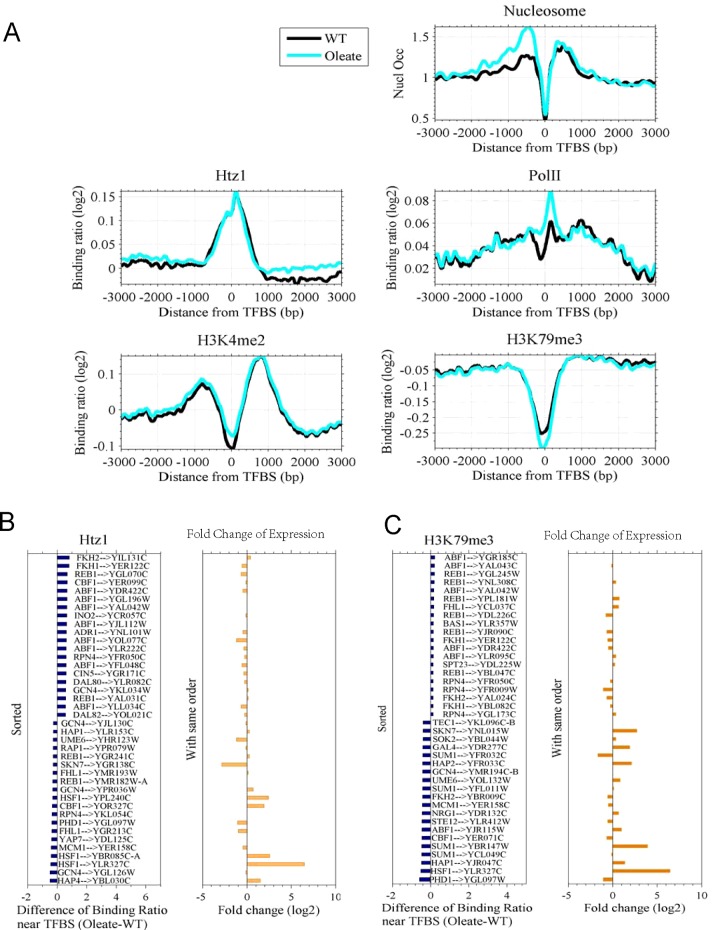
Differences in Htz1 binding and H3K79me3 near TFBSs account for the expression change between unstressed and oleate-stressed cells (**A**) Htz1, Pol II, H3K4me2, H3K79me3 and nucleosome occupancy profiles around TFBSs in unstressed and oleate-stressed cells. (**B**) Differences in the Htz1 binding ratio in the region of −300 bp–+300 bp relative to the centre of the TFBS cluster between unstressed and oleate-stressed cells (left panel) and the expression change (log_2_) for the corresponding genes (target genes; right panel). In the left panel, the name of the transcription factor (TF) and its target genes are shown. Only the 20 top up-regulated and top 20 down-regulated target genes are listed. (**C**) Same as (**B**) except for H3K79me3.

We calculated the difference of the log_2_ binding ratio of Htz1 and H3K79me3 of a 600-bp region of TFBSs in both the unstressed and the stressed conditions and we correlated the difference with changes in the target gene expression downstream of the TFBS (Figures 5B and 5C; Supplementary Figure S7). Decreased and increased Htz1 binding profiles were associated with up-regulated and down-regulated target gene expression respectively (Figure 5B; Supplementary Figure S7A). A similar result was observed for H3K79me3 (Figure 5C; Supplementary Figure S7B). These results indicate that Htz1 and H3K79me3 dynamics near TFBSs are a response to oleate stress and affect gene expression. A strong correlation between expression changes and nucleosome occupancy, Pol II and H3K4me2 dynamics was not observed (Supplementary Figures S7C–S7E).

## DISCUSSION

We investigated the chromatin dynamics in response to oleate stress in *S. cerevisiae*. Oleate stress is caused by a change in the carbon source for *S. cerevisiae*. Oleate inhibits steryl ester synthesis and causes liposensitivity [12]. Our results indicated that citrate cycle-associated genes are enhanced during the glucose-oleate shift. Repression of ribosome genes is frequently suggested in various stresses [6,8]. We also observed the down-regulation of ribosome genes during oleate induction. This indicates that cells up-regulate different genes in response to different stresses, but down-regulate ribosome genes in all types of stress.

Importantly, we suggest that Htz1 is a kind of a sensor for oleate stress. There are four points supporting this hypothesis. First, Htz1 binding between unstressed (wild-type) and stressed conditions is not correlated (Figure 1B). Second, the number of Htz1-bound gene increases upon stress (Figure 4A). Third, Htz1 binding is reduced on up-regulated genes (fold change > 2) (Figure 1D) and increased on ribosome genes, which are down-regulated (Figure 2B). Fourth, the Htz1 dynamics around TFBSs are correlated with transcriptional changes (Figure 5B). Also, Htz1 has a role in the response to heat shock: whenever transcriptionally active Pol II is recruited to promoter regions, Htz1 nucleosomes are displaced; when Pol II departs, H2A.Z nucleosomes return [9]. This suggests that Htz1 can be a stress sensor.

Moreover, we found that nucleosome dynamics are coupled with Htz1 binding changes upon stress. In unstressed cells, nucleosome occupancy is comparable between Htz1-bound genes and Htz1-depleted genes. In stressed cells, the occupancy of Htz1-depleted genes is significantly lower than that of Htz1-bound genes. Considering the fact that only a small fraction (Figure 4A) of Htz1-bound genes loses the Htz1 binding with stress, we inferred that the low nucleosome occupancy of Htz1-depleted genes in stressed cells is not due to Htz1 removal from chromatin. We also suggest that Msn2 has a role in the stress response. Msn2 target genes are enriched for ribosome-related and glucose metabolism pathways (Figure 4D). Additionally, Htz1 binding shows a great change in Msn2 target genes in the stress condition (Figure 4A).

Overall, our analysis highlights the Htz1 binding and nucleosome occupancy dynamics in response to oleate stress.

## CONCLUSION

In the present work, we determined expression change, nucleosome occupancy, Htz1 binding and modifications H3K4me2 and H3K79me3 between unstressed and oleate stressed conditions. The results indicated that citrate cycle-associated genes are enhanced and ribosome genes are repressed during the glucose-oleate shift. We highlighted that Htz1 acts as a sensor for oleate stress. Htz1 binding shows a global change on chromatin and nucleosome dynamics are coupled with Htz1 binding changes upon stress. In oleate stress, the nucleosome occupancy of Htz1-depleted genes is significantly lower than that of Htz1-bound genes. We also found that transcription factor Msn2 is in response to the oleate stress and Htz1 is dynamic in Msn2-target genes.

## Abbreviations

GO: Gene Ontology
H3K4me2: histone H3 lysine 4 dimethylation
H3K79me3: histone H3 lysine 79 trimethylation
KEGG: Kyoto Encyclopedia of Genes and Genomes
TFBS: transcription factor-binding site
TSS: transcription start site
TTS: transcription termination site
